# Supplementary material for: Dense blocks of energetic ions driven by multi-petawatt lasers
Source: Sci Rep. 2016 Feb 29;6:22150. doi: 10.1038/srep22150 (PMC4770588; doi:10.1038/srep22150)
Supplement: Supplementary Information [file srep22150-s3.pdf]

Dense blocks of energetic ions driven by multi-petawatt lasers

S. M. Weng[1,2*], M. Liu[1,2], Z. M. Sheng[1,2,3*], M. Murakami[4], M. Chen[1,2],

L. L. Yu[1,2], and J. Zhang[1,2]

[1]Key Laboratory for Laser Plasmas (Ministry of Education), Department of Physics and Astronomy, Shanghai Jiao Tong University, Shanghai 200240, China

[2]Collaborative Innovation Center of IFSA (CICIFSA), Shanghai Jiao Tong University , Shanghai 200240 , China

[3]SUPA, Department of Physics, University of Strathclyde, Glasgow G4 0NG, UK

[4]Institute of Laser Engineering, Osaka University, Osaka 565-0871, Japan

Correspondence and requests for materials should be addressed to S.M.W. (wengsuming@sjtu.edu.cn) or to Z.M.S. (zhengming.sheng@strath.ac.uk).

Supplementary Figures:

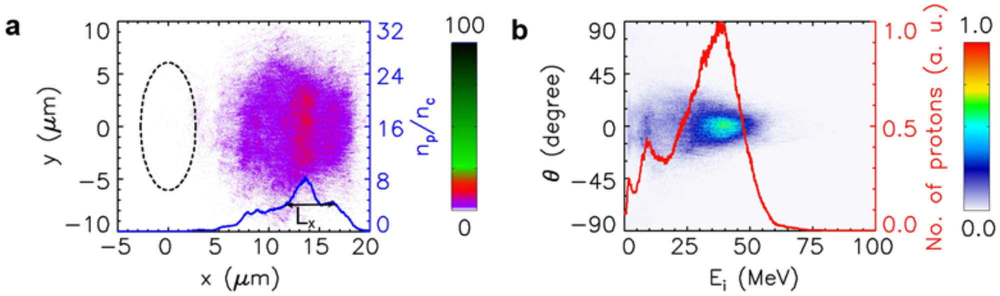

Supplementary Figure S1 | Proton density and energy-angle distributions. (a) Proton distribution (color contour) and density averaged over $|y| \leq 4\,\mu\mathrm{m}$ (blue line) at $t =$ 165 fs in the simulation case using the DM CH_2 target with a substrate. The DM target initially locates in the dashed ellipses, and L_x indicates the instantaneous FWHM dimension in x direction. (b) The corresponding proton energy-angle distribution (color contour) and energy spectrum (red line).

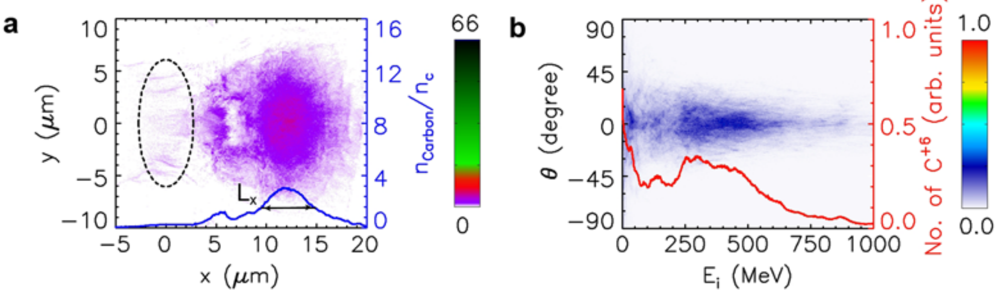

Supplementary Figure S2 | Carbon ion density and energy-angle distributions in the case with a thin substrate. (a) Carbon ion distribution (color contour) and density averaged over $|y| \leq 4\,\mu\text{m}$ (blue line) at $t =$ 165 fs in the simulation case using the DM CH_2 target with a thin (3 μm) substrate. The DM target initially locates in the dashed ellipses, and L_x indicates the instantaneous FWHM dimension in x direction. (b) The corresponding energy-angle distribution (color contour) and energy spectrum (red line).

Legends of Supplementary Movies:

Supplementary Movie S1 | The whole process of ion acceleration. Time evolution of the distribution of carbon ions obtained from the simulation case using the DM CH_2 target with a substrate. The DM CH_2 target initially locates in the dashed ellipses.

Supplementary Movie S2 | Electron motions during the ion acceleration. Time evolution of the distributions of the electrons from the DM part (upper half) and the electrons from the substrate part (lower half) in the simulation case using the DM CH_2 target with a substrate. Both are symmetric about the x-axis, and the dashed ellipse demarcates the initial boundary of the DM CH_2 target. The runaway of heated DM electrons and the return of cold substrate electrons into the DM region are illustrated.
